# Understanding PDE4's function in Alzheimer's disease; a target for novel therapeutic approaches

**DOI:** 10.1042/BST20190763

**Published:** 2019-10-23

**Authors:** Amy J. Tibbo, Gonzalo S. Tejeda, George S. Baillie

**Affiliations:** Institute of Cardiovascular and Medical Science, College of Veterinary, Medical and Life Science, University of Glasgow, Glasgow, U.K.

**Keywords:** Alzheimer's disease, cAMP, cyclic nucleotide phosphodiesterases

## Abstract

Phosphodiesterases (PDEs) have long been considered as targets for the treatment of Alzheimer's disease (AD) and a substantial body of evidence suggests that one sub-family from the super-family of PDEs, namely PDE4D, has particular significance in this context. This review discusses the role of PDE4 in the orchestration of cAMP response element binding signaling in AD and outlines the benefits of targeting PDE4D specifically. We examine the limited available literature that suggests PDE4 expression does not change in AD brains together with reports that show PDE4 inhibition as an effective treatment in this age-related neurodegenerative disease. Actually, aging induces changes in PDE4 expression/activity in an isoform and brain-region specific manner that proposes a similar complexity in AD brains. Therefore, a more detailed account of AD-related alterations in cellular/tissue location and the activation status of PDE4 is required before novel therapies can be developed to target cAMP signaling in this disease.

## Introduction

Phosphodiesterases (PDEs) are the only known enzyme super family that can degrade cyclic nucleotides and their role in cognition was realized in the 1970's following study of a transgenic fly that was deficient in learning [1]. The defective gene was identified as a cyclic-AMP (cAMP) specific PDE [2] which we now recognize as PDE4D [3]. Indeed, there is much literature to suggest that aberrant cyclic AMP (cAMP) signaling is associated with cognitive defects that present in neurodegenerative diseases including Alzheimer's disease (AD). Disease-related errors in signal transduction stem from anomalous PDE function, which results in uncoordinated cAMP responses in certain regions of the brain that can affect memory formation and Aβ production [4].

**Figure 1.**
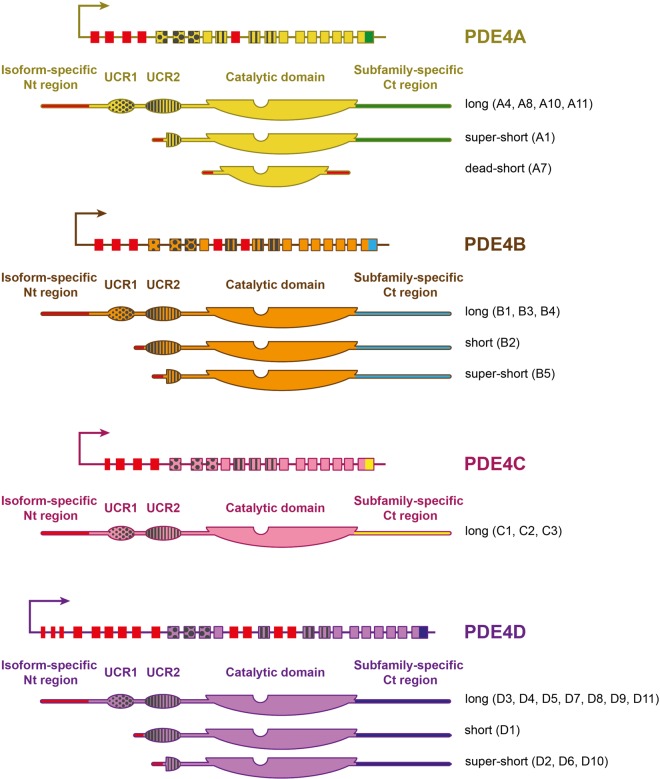
Schematic representation of the four genes of the PDE4 family. Each gene generates multiple isoform variants with unique N-terminal (Nt) regions encoded by distinct specific exons (in red). PDE4 isoforms are classified upon their regulatory regions UCR1 (dots pattern) and UCR2 (line pattern). All isoforms within a specific PDE4 sub-family have identical C-terminal (Ct) regions, except the inactive PDE4A7 that contains a unique 14-residue Ct end.

One family of PDEs that acts to shape cAMP dynamics in neurons and glial cells is PDE4. This family is subdivided into four subfamilies that are encoded by four genes (A, B, C and D) in mammalian cells (reviewed in [5]). All four genes contain upstream exons that undergo splicing to generate a variety of PDE4 isoforms (∼25) in conjunction with the use of different promoters, which contain a unique N-terminal targeting domain, conserved catalytic region and a sub-family specific C-terminal region (Figure 1). Further complexity is derived from Upstream Conserved Regions (UCR1 and UCR2), which act in concert to facilitate enzyme activity changes following PDE4 post-translational modification or dimerization [6].

PDE4s are categorized as long forms (contain UCR1 and UCR2), short forms (contain UCR2) and super short forms (contain a truncated UCR2) [6] (Figure 1). These regulatory domains allow differential regulation of PDE4 activity following modification by phosphorylation and SUMOylation [7]. PDE4s also exist as dimers and this is relevant to the activity status of the enzyme as the UCR1/UCR2 module of one longform partner can occlude the cAMP binding site of the other in a process called ‘trans-capping’ [8]. Modifications such as phosphorylation by protein-kinase A (PKA) (in UCR1) and SUMOylation at the beginning of the catalytic core can lock the PDE4 into the more active (unoccluded) form, whereas phosphorylation by ERK MAP kinase at the end of the catalytic site can promote the inactive dimer conformation (active site occluded) [9].

## PDE4 enzymes orchestrate signaling via CREB

Cognitive enhancement in humans is scarcely achieved, however, it has been noted with the PDE4 inhibitor roflumilast in several preclinical trials, establishing proof of concept that PDE4 is a therapeutic target for AD [10,11]. The potential effects of these inhibitors are attributed to the widely recognized action of cAMP on memory formation [12,13] and cognition [10,11,14,15]. The mechanisms underpinning these functions relate to intracellular increases in cerebral cAMP that activate PKA associated with cAMP response element binding (CREB) protein. CREB activation by PKA is vital for synaptic plasticity and the formation of long-term memory [16,17], hence there has been a lot of interest in agents that enhance phospho-CREB as possible AD therapeutics [18,19]. One strategy that has repeatedly and consistently resulted in protective increases in CREB signaling is the pharmacological inhibition of PDE4 in neurons. Since the 1990s there have been many reports showing that the active-site targeting, PDE4–specific inhibitor rolipram can promote CREB signaling in several brain disease contexts [20–24]. Indeed, it is clear that rolipram reverses learning deficits in rodent models of AD [25,26] via the CREB mechanism [27,28]. As rolipram has equal affinity for all PDE4 isoforms (an attribute that results in side effects that has prevented its clinical use), selective inhibitors that are targeted to PDE4 have been developed to target mainly the PDE4D sub-family of isoforms that are expressed in the hippocampal CA1 region [29,30] and regulate LTP and memory consolidation [31]. One approach has been to develop an allosteric PDE4D selective compound that works by clamping the enzyme in the ‘occluded’ inhibited state [8]. The allosteric PDE4D compound has been shown to promote cognitive benefit in rodent [8], primate models [32], humanized mouse models [33,34] and has shown promising results in human trials. Other PDE4D-directed inhibitors have been designed using slight structural differences between the active sites of PDE4 subfamilies to build in selectivity. The so-called GEBR compounds cross the blood-brain barrier to selectively inhibit PDE4D isoforms, up-regulate CREB signaling and enhance synaptic plasticity and memory formation in rodent AD models [35–39].

## Genetic validation of the role of PDE4D

As already noted, the first learning mutation described in fruit flies is a deletion of the PDE4D gene [2]. PDE4D knock-out mice exhibit memory enhancement and augmented hippocampal CREB signaling that can be mimicked by rolipram treatment or genetic silencing of long-form PDE4D isoforms in wild type mice [40]. RNA interference silencing of longform PDE4Ds can also reverse spatial memory deficits in AD mice that have Aβ infused into their dentate gyrus [41]. Once again, recovery of low cAMP concentrations and attenuated CREB signaling was crucial in the gain of function resulting from PDE4D longform ablation. In further support for the concept that reduced PDE4D activity facilitates cognition and memory formation, genetic mutations in the human PDE4D gene that cause acrodysostosis [42–44] lead to an activation of PDE4D longform enzymes (via PKA phosphorylation) [45] that inhibits CREB activity [46] and promotes intellectual disability [47].

## Investigating the mechanism behind depleted cAMP in AD brains

**Figure 2.**
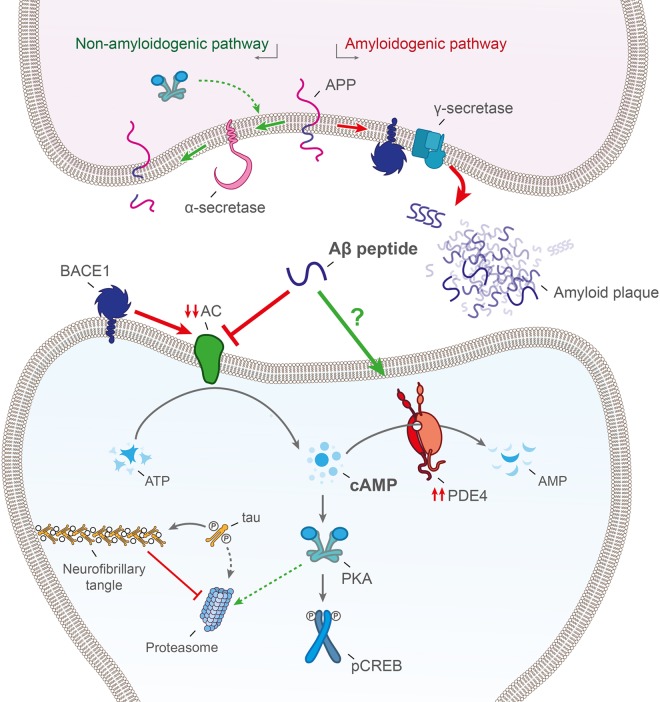
Hypothetical neuronal model of cAMP depletion in AD leading to memory deficits. Aberrant levels of cAMP can be a consequence of an inactivation of AC by Aβ peptide and BACE1 action or a higher activity of PDE4 in neurons. The subsequent decline in PKA action leads to a decrease in proteasomal activity associated with tau accumulation, a down-regulation of CREB signaling and a reduction in Aβ physiological functions.

cAMP is synthesized in neurons by adenylate cyclase (AC) at the membrane and degraded by discretely positioned PDEs that shape cAMP gradients to allow spatially restricted activation of cAMP effectors [48]. Depletion of brain cAMP concentrations in AD [30,49,50] can therefore be a consequence of reduced expression/inhibition of AC or overexpression/activation of PDEs (Figure 2). It has been shown that AC can be inhibited by direct interaction with BACE1 during AD and this promotes reduction in PKA activity and a down-regulation of CREB activation [51] in an Aβ independent fashion. Moreover, a significant reduction in AC expression and activity is observed in the hippocampus of rats that are chronically infused with Aβ [52,53] Interestingly, AC activity measurements from post-mortem AD brains showed a similar decrease in the majority of reports, although contradictory results also exist (reviewed in [50]). For example, one study reported no AC activity changes in the hippocampal region but a 45% decrease in the frontal cortex. The ability of AC to respond to G-protein activation remained unaltered in both areas [54]. Another study described a 50% reduction in basal and stimulated AC activity in post-mortem hippocampi that negatively correlated with amyloid plaque formation. The latter study also pinpointed the foci of signal transduction disturbance at the level of the AC catalytic unit [49]. A third report described a significant decrease in AC activity in AD hippocampi and cerebellum [55]. Conversely, the cognitive function improvement in AD transgenic mice after pituitary adenylate cyclase-activating polypeptide (PACAP) treatment [56] possibly occur via AC, as its activation by forskolin prevents pathological Aβ inhibition of LTP [56]. Moreover, it has been shown that the cAMP/PKA pathway can induce production of Aβ in physiological conditions which is instrumental for the switching of E-LTP to L-LTP through PDE4 inhibition [57]. Therefore, memory deficits in AD could be caused by a dysregulated cyclic nucleotide signaling that leads to loss of physiological function of Aβ within the brain including synaptic plasticity [4,58,59].

Currently, therapeutic treatment with the allosteric PDE4D inhibitors Gebr-7b and Gebr-32a improved cognition in the APP/PS1 mouse model but was ineffective at reducing Aβ load in the hippocampus [36,39]. Reciprocal results were seen with the use of rolipram, although there was increased phosphorylation of CREB reversing the deficit present in AD [25]. Interestingly, rolipram led to the clearance of aggregated tau in the frontal cortex in mouse models of tauopathy [60]. *In vitro* studies identified that increasing proteasomal activity through cAMP/PKA/pCREB resulted in a noted decrease in the levels of ubiquitin conjugates suggesting that PKA induction is responsible for the enhanced tau clearance [61]. Treatment with rolipram in mice throughout early disease stage was found to promote proteasomal activity and lead to a reduction in tau accumulation with subsequent improvement in cognitive defects [61,62]. Thus, the interplay between cAMP, Aβ and tau protein adds further levels of complexity to an already intricate pathway.

Surprisingly, in the light of the fact that there is a large body of literature unequivocally supporting use of PDE4 inhibitors as a therapy for memory/cognition enhancement in AD, very little work has been done to profile PDE4 changes during disease progression. Such data is important to enhance our understanding of why this enzyme family is so pivotal for AD. Of particular importance has been the sub-family PDE4D (reviewed in [63]). A few studies have attempted to determine whether PDE4 expression is altered in AD brains. In post mortem, human hippocampi, TaqMan Gene Expression profiling of the nine human PDE4D isoforms (PDE4D1 to 9, inclusive) was evaluated and all were found to be expressed in both healthy and diseased brains (*n* = 3 and *n* = 1, respectively) [30]. However, in the AD hippocampus, expression of the majority of the isoforms, except for PDE4D1,PDE4D2 and PDE4D4, was dramatically reduced [30]. A different study using RT-PCR techniques also highlighted no overall change in PDE4D in the temporal cortex of human AD brains [64], which can be the result of a net effect of all the isoforms or regional differences in expression in the brain. In conjecture with lack of PDE4D change, both PDE4A and PDE4B mRNA [65] was increased in the entorhinal cortex. With respect to PDE4 protein, increases in the expression of PDE4A, B and D long forms (using Western blotting) have been described in mice hippocampi following infusion with Aβ^1–42^ [66,67]. From the data that exists there seems to be a discrepancy between the obvious utility of PDE4 inhibitors in AD and the lack of evidence in human brains that PDE4 level change during disease progression. Crucial to this conceptual problem is the dearth of information on PDE4 activity changes in AD brains. Amounts of PDE4 mRNA do not always correlate to protein levels and western blotting cannot always evaluate the activity state of PDE4s, which as stated earlier can be activated and inhibited by point mutations [44], post-translational modification [7,68,69] or by direct association with protein partners [70] or other binding molecules such as phosphatidic acid [71].

## Understanding molecular changes in cAMP signaling that underpin disease progression is vital to the development of new treatment regimes

A major caveat of targeting PDE4D for cognitive intervention is the vast diversity of the sub-family isoforms, each with unique expression patterns, interacting partners and specific roles within the cell. For example, β1AR is known to selectively interact with PDE4D8 [72] whereas β2AR has a higher affinity for PDE4D5 [73]. The β1AR/PDE4D8 complex is only present during the absence of agonist binding allowing for the modulation of cAMP and subsequently PKA activation in the local vicinity [72]. This control is lost upon ligand binding. The reverse is true for the β2AR/PDE4D5 complex, which is only present after recruitment of β-arrestin. It is through this mode of action that β2AR switches between activation of ACs and activation of extracellular signaling [73]. Then, an incorrect inhibition of either or both isoforms through broad PDE4D inhibition could lead to signaling dysregulation and undesirable outcomes. Therefore, it is becoming clear that an increase in the specificity targeting of PDE4D isoforms will be necessary to improve efficacy while diminishing the numerous side effects, including emesis and headaches [74], that have plagued the current PDE inhibitors.

Novel complex-specific PDE4D therapeutics for AD can only be developed by a deep characterization of the underlying mechanisms of the disease and its progression. Thus, the ideal target candidate would be a PDE that is pathologically overexpressed in the tissue of interest and responsible for the dysregulated cyclic nucleotides signaling. An example that supports this view is the differential improvement in working memory experienced after rolipram treatment in young but not old monkeys [75]. The lack of cognitive enhancement correlates to a decline in PDE4 expression in the striatum and cortex with aging [76]. In order to avoid the overstimulation of an already disinhibited cAMP pathway, an exhaustive comparative analysis of cAMP and PDE4 mRNA, protein and activity from diseased and healthy tissue/cells is required. This methodology has been previously successful in prostate cancer, where transcripts for PDE4D long forms (and in particular PDE4D7) are abundant in androgen-sensitive cancer stages but practically disappear in androgen insensitive cells that are metastatic and drive disease progression [77]. The change in mRNA corresponds to a paucity of PDE4D7 protein and activity that increases cAMP signaling. These changes are so reproducible that PDE4D7 is now regarded as an important biomarker that can predict pre and post-surgical risk in patients, which allows better treatment choices to be made [78,79]. In another example, namely autosomal dominant polycystic kidney disease (ADPKD), a comparable situation arises where chronically elevated cAMP [80] resulting from activation of AC [81] and reduced levels of PDE4C [82,83] drives cyst formation. Here, a novel PDE4 compound has been developed to suppress excess cAMP by allosterically activating PDE4 longform [84]. In human and animal models of ADPKD, pharmacological activation of PDE4 puts a brake on cAMP signaling and profoundly inhibits cyst formation.

## Conclusion

Both cases outlined above illustrate the need for a deeper understanding of the molecular ‘fingerprint’ of cAMP signaling in AD. The effectiveness of inhibiting the PDE4D sub-family by pharmacological means or genetic silencing suggests that this enzyme has a unique coordinating role in cognition that is maladapted during AD. Further analysis of the identity and precise cellular location of single isoforms of PDE4D as well as the activation state changes that occur during disease should allow novel therapeutic approaches to be developed.

**Perspectives**

- Importance: PDE4 inhibitors have been shown to be effective in enhancing the cognition and memory in AD but little is known about changes in PDE4 activity during the disease. This review looks at mechanistic evidence as to why PDE4 may be a viable target in AD and suggests that more information on the identity, amounts and activation states of PDE4 isoforms in AD brains may help influence future treatments.
- Summary of current thinking: Cyclic AMP in the brain has long been thought to promote memory formation and enhance cognition. Many reports using a variety of techniques have shown that specific inhibition of PDE4, and specifically PDE4D, leads to an increase in cAMP which in turn promotes the activation of PKA, leading to the phosphorylation of CREB. Active CREB signaling in the brain is vital for synaptic plasticity and the formation of long-term memory and hence is a therapeutic target for AD.
- Comment on future directions: The paucity of information surrounding the changes in PDE4 levels and activation state that occur in AD currently do not correlate with the abundance of evidence suggesting that this enzyme family is a prime target for therapeutic intervention. Precise information about individual isoforms, their cellular/tissue distribution and activation state is required to better tailor current PDE4 inhibition strategies for AD.

## Abbreviations

AC: adenylate cyclase
AD: Alzheimer's disease
ADPKD: autosomal dominant polycystic kidney disease
PACAP: pituitary adenylate cyclase-activating polypeptide
PDEs: Phosphodiesterases
PKA: protein-kinase A
